# Optimizing Clinical Decision Support System Functionality by Leveraging Specific Human-Computer Interaction Elements: Insights From a Systematic Review

**DOI:** 10.2196/69333

**Published:** 2025-05-06

**Authors:** Ali Azadi, Francisco José García-Peñalvo

**Affiliations:** 1 GRIAL Research Group Computer Science Department Universidad de Salamanca Salamanca Spain

**Keywords:** human-computer interaction, clinical decision support system, usability, user-centered design, artificial intelligence

## Abstract

**Background:**

Clinical decision support systems (CDSSs) play a pivotal role in health care by enhancing clinical decision-making processes. These systems represent a significant advancement in medical information systems. However, optimizing their effectiveness requires accounting for various human-computer interaction (HCI) elements that influence their functionality and user acceptance.

**Objective:**

This study aimed to identify and categorize key HCI elements that impact CDSS performance to enhance system usability, adaptability, and decision-making accuracy.

**Methods:**

We conducted a systematic literature review, identifying 923 studies from the databases PubMed, Scopus, and Web of Science. Papers were screened and selected based on predefined inclusion criteria. A rigorous quality assessment process was applied to ensure the relevance and reliability of the included studies. Ultimately, of the 923 papers identified, 43 (4.7%) that specifically addressed HCI elements applicable to CDSS environments were included in the final analysis. Data extraction and synthesis were performed to answer the research questions regarding HCI elements.

**Results:**

A total of 12 distinct HCI elements were identified, each with the potential to influence CDSS functionality. These elements align with the International Organization for Standardization (ISO) 9241-11 framework, which defines usability in terms of effectiveness, efficiency, and satisfaction. “User satisfaction,” “flexibility,” and “individuality” enhance satisfaction by improving system adaptability and user acceptance. “Visibility,” “explainability,” and “user control” strengthen effectiveness by supporting decision-making and error prevention. “Ease of use” improves efficiency by streamlining interactions and reducing cognitive load. Some elements influence effectiveness and efficiency, such as “data entry,” which ensures structured inputs for decision accuracy while optimizing workflows. Likewise, “alerts” provide timely information for effective decision-making and, simultaneously, are designed to avoid overwhelming users and maintain system efficiency. “Simplification” and “mental effort” also optimize workflows and reduce complexity. Furthermore, “interface” impacts effectiveness and efficiency by supporting accurate decision-making and streamlining user interaction. This categorization, aligned with ISO 9241-11, underscores the context and task dependency of usability, highlighting that HCI elements must be adapted to different user needs and environments for effective clinical decision-making.

**Conclusions:**

This study addresses a critical gap in CDSS research by offering a comprehensive framework of HCI elements tailored to the CDSS environment. Incorporating these elements into system design can improve user satisfaction, reduce data errors, and enhance the accuracy of medical decisions. The findings lay the groundwork for future research, offering practical guidelines for developing more reliable and efficient CDSS systems in medical informatics fields.

## Introduction

### Background

Clinical decision support systems (CDSSs) provide clinicians with computer-generated clinical knowledge and patients with relevant health information presented at the right time to support optimal patient care [1]. Poorly designed user interfaces (UIs) may impede structured data entry, potentially compromising data quality and resulting in incomplete datasets [2]. Such shortcomings can negatively impact the effectiveness of integrated medical information systems when used within a CDSS framework [3]. CDSSs, as a prominent method for enhancing clinical decisions effectively, rely on patient-entered data in electronic medical records (EMRs). Data scientists face challenges in analyzing data obtained from EMRs due to the prevalence of errors, including typos and incorrect entries. In other words, incomplete medical advice originates from incomplete and unstructured prepared data for CDSSs [4]. Physicians often express these errors as stemming from an inadequate interaction with the system [5]. Human-computer interaction (HCI), based on the solid principles of ergonomics, cognitive science, and psychology, is pivotal in creating practical and beneficial technology for all users [6]. Indeed, HCI science advocates for physicians through technological solutions and enhances their comprehension of the significance of their daily processes and the subsequent use of data stored in electronic health records (EHRs) [7].

Although the HCI concept typically encompasses how computer systems are designed for ease of use, efficiency, and effectiveness [8], HCI in the medical realm implies the investigation and design of computer systems that expedite productive and intuitive interaction between physicians and technology to boost user experience (UX) and achieve the desired result [9].

Neglecting HCI elements in medical systems can lead to inadequate graphical UIs, potentially hindering physician adoption and efficacy within the digital health care landscape [10]. Conversely, some studies have demonstrated that incorporating HCI elements improves user acceptance and enhances the effective use of CDSS functionalities [11]. Despite several studies addressing HCI elements and their components, the lack of studies on HCI elements that can affect CDSS performance is quite noticeable. In this regard, related concepts, such as usability, UX, or user-centered design (UCD), all of which contribute to physician satisfaction, need to be investigated. HCI assists in identifying system requirements, including text style, fonts, layout, graphics, and color. Meanwhile, usability focuses on efficiency, effectiveness, utility, ease of learning, and ease of assessment. Combining these perspectives ensures a comprehensive approach to designing a CDSS that meets technical and user-centric demands [12].

The necessity of distinguishing HCI elements specifically for CDSSs, in comparison with decision support systems (DSSs), is derived from the unique demands of the health care environment [13]. CDSSs must seamlessly integrate complex medical knowledge, ensure adherence to stringent clinical guidelines, and accurately handle sensitive patient data, all while minimizing the cognitive load on health care professionals [14]. These systems support clinical decisions that directly impact patient outcomes. This is why elements of usability and UX must be considered. Unlike generic DSSs, CDSSs must navigate the intricate nature of medical decision-making**.** Hence, a tailored approach to HCI elements is needed to address these specialized requirements effectively [15]. In addition, EMRs must be designed using comprehensive HCI elements to serve as a gateway to the data required by the CDSS. Poorly designed HCI in EMRs can lead to improper data entry or retrieval [16]. These errors can have serious consequences, potentially impacting patient safety and even leading to loss of life. Conversely, well-designed HCI can facilitate accurate data handling, improving patient care and significantly enhancing outcomes [17].

Due to the absence of targeted studies that focus on leveraging HCI elements within the CDSS environment, conducting this research is crucial. By systematically identifying and evaluating practical HCI elements specific to CDSSs, this study provides a comprehensive guide to enhance the performance and usability of these systems. This focused investigation offers a unique framework that health care professionals and system developers can use to implement more effective and user-friendly CDSS solutions. The scientific contribution of this study lies in providing a structured methodology to improve CDSS performance, ensuring better integration of HCI elements tailored to the health care context.

### Systematic Literature Review

Conducting a systematic literature review (SLR) [18] was the most effective method for this research because it allowed for a rigorous and comprehensive collection of existing studies in the field. An SLR provides a transparent and replicable process for identifying, evaluating, and synthesizing relevant literature. Using the SLR approach, we endeavored to extract and categorize HCI elements that are especially applicable within the CDSS environment, ultimately paving the way for improved CDSS performance.

On the basis of the concerns highlighted in this study, we formulated the following research questions (RQs):

- RQ1: Which HCI elements in medical information systems can affect CDSS functionality?
- RQ2: How does incorporating the identified elements improve CDSS performance?

By seeking a response to the first RQ, we aimed to identify and extract HCI components that have the potential to affect CDSS functionality, as pointed out in the reviewed papers. The second RQ focuses on investigating how the identified HCI elements, when applied within medical environments and information systems, contribute to enhancing CDSS performance.

The remainder of this work is organized as follows. The Methods section describes the methodology and the review steps. The Review Planning subsection details the SLR planning phase, while the Review Process subsection presents the review and data extraction steps. The Results section presents the findings from the analyzed studies to answer the RQs. Finally, the Discussion section presents the principal findings, with some clues and future research lines outlined in the Conclusions subsection.

## Methods

### Overview

This study followed the SLR methodology outlined by Kitchenham [19] and Kitchenham and Charters [20] to ensure a thorough and unbiased synthesis of existing research. The SLR was complemented by a systematic literature mapping following the method proposed by Kitchenham et al [21]. This segment outlines the protocol used to perform the SLR, providing the necessary information to support the subsequent findings. According to the guidelines provided by Kitchenham [19] and Kitchenham and Charters [20], the SLR process consists of 3 principal phases: planning, conducting, and reporting the study. We adhered strictly to the defined SLR protocols and methodological nuances articulated in the study by Pati and Lorusso [22] to ensure transparency in both the research process and results.

Before planning this SLR, a preliminary search was conducted to ascertain that no recent SLR had been undertaken to identify HCI elements within CDSSs. This preparatory appraisal involved searching various electronic databases, including Scopus, Web of Science, and PubMed, using terms related to the methodology (eg, *SLR* or *systematic literature review*) and the review focus (eg, *HCI*, *usability*, *user experience*, *elements in CDSS*, and equivalent concepts). The results of this preliminary search confirmed the absence of an SLR on the specified theme, thereby justifying the implementation of this review to fill the identified research gap.

### Review Planning

The review planning process involves identifying and defining various dimensions to provide the foundation for executing the review, such as formulating the RQs, detailing the protocol followed, and any other relevant information to ensure the traceability of the review process. We outline these dimensions in this subsection.

#### Mapping Questions

This study’s main concerns and RQs are outlined in the Introduction section. To further explore the context and breadth of our SLR, we formulated 4 mapping questions (MQs) to guide our analysis:

- MQ1 (geographic distribution of studies): Where have studies related to HCI elements in CDSSs been conducted worldwide?
- MQ2 (year-wise distribution of studies): What is the distribution of studies by identified HCI element over the years?
- MQ3 (publication venues): What is the distribution of studies between journal papers and conference papers?
- MQ4 (focus distribution): What percentage of the identified studies discuss each HCI element (eg, alerts, interface, system design defects, and mental effort)?

It is important to note that MQ4 (focus distribution) does not have a dedicated subsection. Instead, it has been addressed throughout the identification and discussion of each HCI element. By addressing these MQs, we aim to provide a comprehensive overview of the current research landscape, highlighting key trends and patterns that inform the influence of HCI elements on CDSS performance and user interaction.

#### Population, Intervention, Comparison, Outcome, and Context Framework

Regarding the questions posed, the population, intervention, comparison, outcome, and context method proposed by Petticrew and Roberts [23] (outlined in the following subsections) and which provides a structured framework for research reviews was used to define the review scope.

##### Population

We considered all studies that involved software solutions, regardless of their implementation or design. Although the review focuses on the properties and attributes of EMRs, the target studies addressed HCI elements within CDSSs.

##### Intervention

The interventions of interest included those that suggested or introduced some elements addressing HCI issues in EMRs or eHealth systems, excluding stand-alone solutions such as mobile health apps.

##### Comparison

No comparison between interventions was planned.

##### Outcome

The fundamental outcome of this review is to identify key HCI elements that can enhance the functionality of CDSSs.

##### Context

We considered all studies relevant to EMR and eHealth systems. The review includes all papers describing the successful implementations and designs of EMRs in medical environments, such as hospitals and medical centers worldwide.

#### Inclusion and Exclusion Criteria

After defining the review’s scope, we established inclusion and exclusion criteria (Textbox 1) to choose pertinent literature for addressing the RQs.

**Inclusion and exclusion criteria.**

**Inclusion criteria**

- The paper proposes a pragmatic and implementable solution (eg, method, technique, model, tool, or framework).
- The proposed solution is applied to software, applications, platforms, services, infrastructures, or systems.
- The study focuses on electronic medical records, electronic health records, or eHealth.
- The proposed solution supports or addresses the tailoring of attributes and criteria to improve medical decision support capabilities.
- The paper is written in English.
- The paper was published between 2000 and July 2024 (when the search queries were executed).
- The full paper is available.
- The tailoring capabilities relate to human-computer interaction, usability, user interface, user experience, or user-centered design.
- The article is published in a peer-reviewed journal, book, or conference (including only conferences ranked B or higher in the CORE Conference Ranking list or classified in the top 2 quartiles in the Scimago Journal Rank index).

**Exclusion criteria**

- The paper does not propose a pragmatic and implementable solution (eg, method, technique, model, tool, or framework).
- The proposed solution is not applied to software, applications, platforms, services, infrastructures, or systems.
- The study is not focused on electronic medical records, electronic health records, or eHealth.
- The proposed solution does not support or address the tailoring of attributes and criteria to improve medical decision support capabilities.
- The paper is not written in English.
- The paper was published before 2000 or after July 2024 (when the search queries were executed).
- The full paper is not available.
- The tailoring capabilities do not relate to human-computer interaction, usability, user interface, user experience, or user-centered design.
- The article is not published in a peer-reviewed journal, book, or conference (including only conferences ranked B or higher in the CORE Conference Ranking list or classified in the top 2 quartiles in the Scimago Journal Rank index).

#### Search Strategy

It is imperative to identify the most significant databases regarding the search domain in which the queries will be executed to obtain relevant outcomes from the investigation. For this study, 3 electronic databases were selected: Scopus, Web of Science, and PubMed. These databases were chosen based on the following criteria:

- They serve as reference databases in the research domain.
- They are highly relevant to the study context.
- They support the use of search strings and Boolean operators to augment the results of the retrieval process.

On the basis of the research context used to construct the search query, the following terms were included:

- Usability—this concept refers to efficiency, effectiveness, and physician satisfaction [24,25]. It emphasizes maximizing system facilitators to enhance usability [26,27].
- All terms related to HCI—HCI involves interaction and communication between users and computer systems, encompassing the exchange of information, symbols, and actions to facilitate seamless interaction between humans and computers [28]. In this regard, *HCI elements* are defined as the specific elements or attributes that influence user interaction and experience within the determined framework [29]. *HCI elements* and *HCI factors* in medical settings highlight the importance of UCD, ensuring that medical staff are integrally involved in developing and refining medical systems for enhanced performance and satisfaction [30,31]. The terms *HCI factors* and *HCI elements* are sometimes used interchangeably in HCI literature. *HCI factors* often refer to broader influences on usability, while *HCI elements* represent specific design components.
- User-centered design (UCD)—UCD emphasizes the essentiality of considering end users’ viewpoints in shaping or evolving a product or software, predominantly based on end-user feedback [32].
- User interface (UI)—in medical information systems, the UI serves as the direct interface between the system and physicians. It can be categorized as static, appearing the same for all users; or dynamic, adapting to varying circumstances based on user interactions with the system [33].
- CDSS—CDSSs encompass a broad range of tools and interventions, both computerized and noncomputerized, to aid clinicians in their complex decision-making processes [34,35]. They are being integrated into EMRs and computerized clinical workflows worldwide, empowering health care providers to access timely and pertinent information, ultimately leading to better patient outcomes through informed decision-making during clinical care [14].
- Artificial intelligence (AI)—in medicine, AI refers to the ability of medical systems to respond to environmental factors and support decision-making, achieving results comparable to human medical professionals [36]. CDSSs that use AI function as digital dynamic knowledge systems that leverage patient data to create tailored recommendations for clinicians [37]. In other words, CDSSs are meant to assist rather than perform clinical decision-making [38].

In line with the research goals, we only investigated HCI elements that directly or indirectly impact systems designed for medical decision-making. Hence, HCI elements not relevant to this context were excluded from consideration.

#### Quality Assessment

##### Overview

Although inclusion and exclusion criteria help identify relevant works for a study, they do not address the quality of the papers retrieved regarding their ability to address the RQs. Therefore, we developed a separate set of quality assessment criteria. We adopted a scoring system in which each coauthor independently evaluated the studies using the quality questionnaire proposed by Kitchenham and Brereton [39]. The scoring was as follows: a “yes” response earned 1 point, a “partially” response earned 0.5 points, and a “no” response earned 0 points. The maximum score a study could receive was 7 points. The quality questionnaire and its corresponding assessment phases are detailed in the following subsections.

##### Design Clarity and Relevance

Are the research aims and objectives clearly stated and aligned with the study’s focus on HCI elements in CDSSs?

##### Data Collection and Relevance

Does the paper provide comprehensive and relevant data pertinent to HCI elements in CDSSs, including metrics for evaluation?

##### Empirical Measurement and Methodology

Are the HCI elements empirically measured with well-defined metrics? Is the methodology for evaluating these elements clearly described?

##### Analysis and Documentation

Are the research results documented with granularity, including participant information, observational units, and analysis methods?

##### Conclusion Validity and RQs

Do the study’s conclusions adequately answer the RQs? Are the implications for HCI and CDSSs clearly articulated?

##### HCI Evaluation Method

Is the HCI evaluation method used in the study justified and described in sufficient detail?

##### Relationship Between HCI Elements and CDSS Outcomes

Does the study adequately illuminate the relationship between the applied HCI elements and the CDSS outputs or results and their consequences?

##### Quality Assessment and Interrater Reliability

The maximum score a study could receive was 7 points. To advance to the next phase of the review, studies were required to score at least 5 out of 7 points. To validate the quality assessment results and demonstrate process consistency, we computed a measure of interrater reliability using Krippendorff α [40]. The Krippendorff α value was 75.33%, indicating that the data were interpreted similarly and acceptably among the coauthors. When discrepancies arose, we engaged in discussion sessions to reach a consensus.

#### Query Strings

##### Overview

The search strings for each considered source were generated using relevant search terms derived from the population, intervention, comparison, outcome, and context methodology outcomes [41], connected by Boolean “and” or “or” operators. The canonical search equation provides a standardized template that ensures consistency across different databases. This canonical search equation is then adapted to fit the specific syntax of each database. The canonical search equation is as follows: (“human-computer interaction” OR “HCI” OR “usability” OR “user experience” OR “user interface” OR “user-centered”) AND (“electronic medical records” OR “EMR” OR “electronic health records” OR “EHR”) AND (“clinical decision support systems” OR “CDSS” OR “decision support system*” OR “DSS” OR “artificial intelligence” OR “AI”).

Using the canonical search equation, we formulated the following queries tailored to each of the 3 selected databases to retrieve all relevant studies, including those addressing the implications of physicians’ interactions with systems. Each database required distinct syntax, as outlined in Textbox 2.

**Search syntax for each database.**

**Scopus**

(TITLE-ABS-KEY (“human-computer interaction” OR “HCI” OR “usability” OR “user experience” OR “user interface” OR “user-centered”) AND TITLE-ABS-KEY (“electronic medical records” OR “EMR” OR “electronic health records” OR “EHR”) AND TITLE-ABS-KEY (“clinical decision support systems” OR “CDSS” OR “*decision support system*” OR “DSS” OR “artificial intelligence” OR “AI”))

**Web of Science**

(TS= (“human-computer interaction” OR “HCI” OR “usability” OR “user experience” OR “user interface” OR “user-centered”) AND TS= (“electronic medical records” OR “EMR” OR “electronic health records” OR “EHR”) AND TS= (“clinical decision support systems” OR “CDSS” OR “decision support system*” OR “DSS” OR “artificial intelligence” OR “AI”))

**PubMed**

((“human-computer interaction” OR “HCI” OR “usability” OR “user experience” OR “user interface” OR “user-centered”) AND (“electronic medical records” OR “EMR” OR “electronic health records” OR “EHR”) AND (“clinical decision support systems” OR “CDSS” OR “decision support system*” OR “DSS” OR “artificial intelligence” OR “AI”))

##### Limiting Retrieved Results

Each database required distinct syntax, as outlined in the query strings presented above. In subsequent stages of this review, the retrieved results were limited to those that had the potential to address the predefined RQs.

### Review Process

The PRISMA (Preferred Reporting Items for Systematic Reviews and Meta-Analyses) guidelines [42] were followed for reporting this SLR. Refer to Multimedia Appendix 1 for the PRISMA checklist.

The data-gathering process was segmented into several stages, during which various inspections were performed.

Once the search was completed on July 25, 2024, the paper selection process was conducted in the following stages:

1. The raw data, consisting of records retrieved from the 3 databases, were compiled into a Zenodo repository (Multimedia Appendix 2). This dataset, which contains data from 923 papers, is organized into separate sheets for each database: Scopus (n=506, 54.8%), Web of Science (n=188, 20.4%), and PubMed (n=229, 24.8%).
2. After arranging the records, duplicate papers were eliminated. Of the 923 papers, 326 (35.3%) were removed, leaving 597 (64.7%) for the next stage.
3. At the first step of the screening, reading the titles, abstracts, and keywords and applying the inclusion and exclusion criteria resulted in 444 (74.4%) of the 597 papers being excluded, leaving 153 (25.6%) for the next phase.
4. Of these 153 papers, the full text of 5 (3.3%) was not accessible; hence, we read in detail and further scrutinized 148 (96.7%) papers. After an exhaustive examination of these 148 papers, we removed 105 (70.9%) for the following reasons: 49 (46.7%) involved HCI elements in medical environments, but these elements were unrelated to CDSSs; 38 (36.2%) did not explicitly focus on HCI or usability elements, although they discussed CDSS functionality; and 18 (17.1%) did not clearly explain the relationship between the identified HCI elements and CDSS functionality.
5. Ultimately, of the initially identified 923 papers, 43 (4.7%) were selected for the final analysis and review (Multimedia Appendix 3 [7,43-84]).

## Results

### Overview

This section provides a detailed analysis of the findings derived from our SLR on HCI elements that influence CDSSs. By thoroughly examining the 43 articles included for analysis, we aimed to answer the formulated RQs and MQs, thereby delineating the boundaries of this research area.

Our RQs focused on identifying the HCI elements that influence medical data management and CDSS functionality (RQ1) and understanding how these identified HCI elements can impact CDSS performance (RQ2). These questions were essential for understanding the relationship between HCI elements and the efficiency, accuracy, and usability of CDSSs.

In addition to these RQs, we formulated several MQs to comprehensively explore the scope and context of the studies. These included understanding the geographic distribution of research efforts in this area, analyzing the temporal distribution of the included studies, and identifying their publication venues. Furthermore, we examined the topic distribution in this SLR to determine the percentage of attention each identified HCI element received across the reviewed studies.

This analysis provides insights into the HCI elements investigated in the studies and how they influence CDSS functionality. This comprehensive overview offers valuable information on how HCI elements can be optimized to enhance the overall performance and effectiveness of CDSSs, ultimately contributing to improved patient care and decision-making within medical settings.

**Figure 1:**
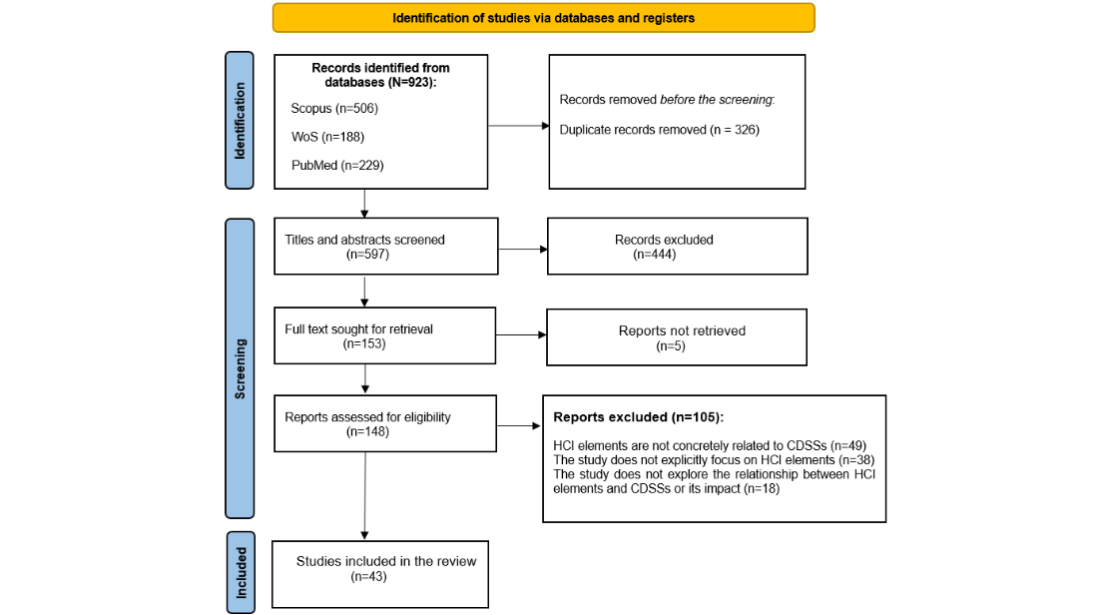
PRISMA (Preferred Reporting Items for Systematic Reviews and Meta-Analyses) 2020 flow diagram showing the number of studies identified, screened, assessed for eligibility, and included in the final analysis. CDSS: clinical decision support system; HCI: human-computer interaction; WoS: Web of Science.

The PRISMA flow diagram [42] illustrates the steps taken to extract the required data (Figure 1).

### Geographic Distribution of Studies

**Figure 2:**
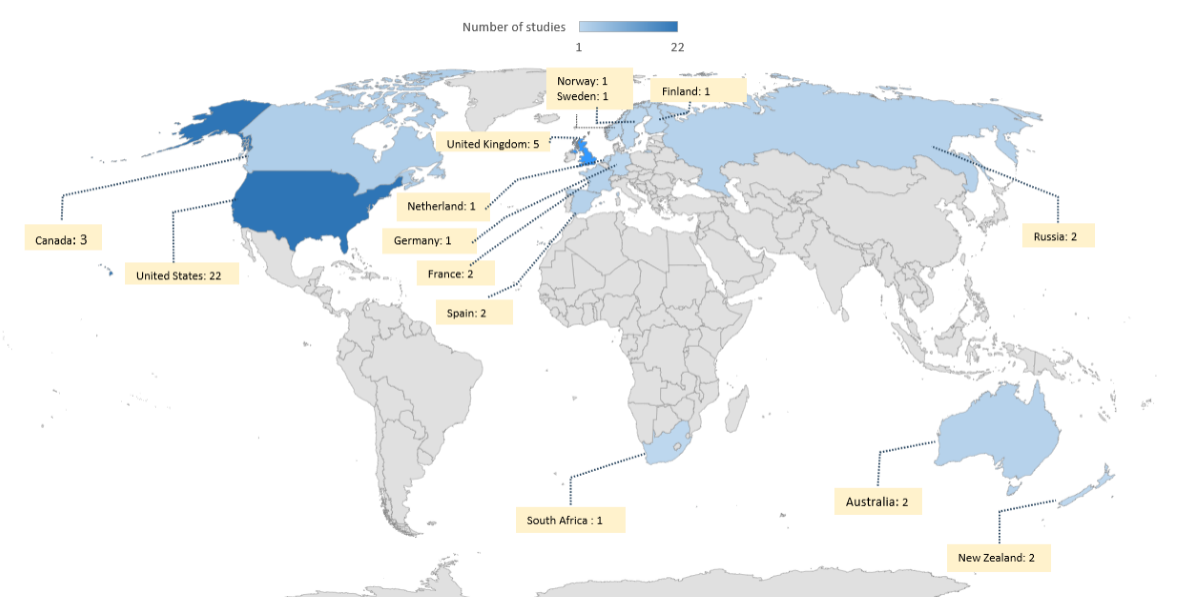
Geographic distribution of the included studies.

Our investigation to address MQ1 was carried out by considering the affiliations of all authors. Consequently, a study with authors from Spain, the United Kingdom, and Sweden was attributed to all 3 countries. In total, 43 studies were conducted across 14 countries. As depicted in Figure 2, the geographic distribution reveals a significant concentration of research in the United States and Europe, reflecting global interest in enhancing CDSSs through improved HCI elements. The United States contributed the most publications, with 51% (22/43) of the relevant studies, followed by the United Kingdom with 12% (5/43); Canada with 7% (3/43); and Russia, Spain, France, and Australia with 5% (2/43). The remaining countries—Sweden, South Africa, Norway, New Zealand, Finland, and Germany—each accounted for 2% (1/43) of the studies.

Despite the rapid technological advancements and growing health care needs in Asia, our analysis revealed a notable lack of studies relevant to HCI elements in the CDSS realm from Asian countries. Addressing this discrepancy is crucial for developing cultural and contextual usability solutions to enhance CDSS performance.

Our exploration indicates that the emphasis on distinct HCI elements varies by country based on specific demands, policies, and priorities. In the United States, which has the highest research activity in this field, studies have predominantly investigated HCI elements related to CDSS alerts (9/22, 41%), UI (3/22, 14%), and ease of use (2/22, 9%). Although these core HCI elements are imperative, some specific HCI demands may be considered depending on cultural differences and local needs [85].

### Year-Wise Distribution of Studies by Identified HCI Elements

**Figure 3:**
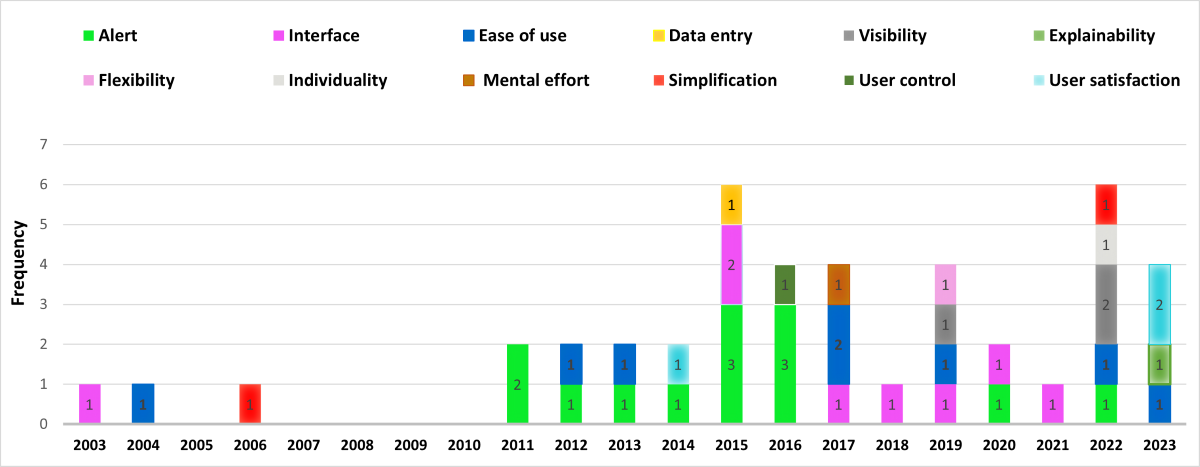
Scattering leverage of human-computer interaction elements in clinical decision support systems over the years.

Through the review process, we identified 12 HCI elements. Each included paper primarily focuses on 1 main element, as shown in Figure 3. While some papers discuss multiple HCI elements, the figure presents the dominant HCI element addressed in each paper. The bar graph illustrates which HCI elements related to CDSS received attention each year. Although the gradual formation and evolution of CDSS technology began in 1990 [14], HCI elements have gained significant attention in medical settings since the 1990s, with increasing emphasis on usability, UX, and interface design in health care technology [86]. This study depicts the conjunction of 2 concepts—HCI and CDSS functionality—that have emerged since 2003 and have seen significant advances, especially since 2015. Figure 3 highlights this growth, which is tightly related to MQ2. As shown in Figure 3, an investigation gap existed in this realm between 2006 and 2011, and most studies on this subject were carried out in 2015 and 2022. Since 2011, there has been a growing interest in exploring the relationship between HCI elements and CDSS functionality.

This trend reflects the increasing comprehension of the importance of UCD in health care technology, aiming to improve the usability and effectiveness of CDSSs in supporting clinical decision-making processes [87].

### Publication Venues: Distribution by Study Type (Journal, Conference, or Book Chapter)

Nearly all of the included papers (42/43, 98%) were published in academic journals. This indicates a strong preference for disseminating research findings related to HCI elements in CDSSs through journal publications, which are often peer reviewed and highly regarded in the academic community. Moreover, 1 (2%) of the 43 papers was a book chapter, which provides comprehensive overviews and in-depth discussions on specific topics, contributing valuable insights to the field.

This distribution, as the explicit answer to MQ3, highlights the credibility and rigor of the selected studies, ensuring that the insights and conclusions drawn from this review are thoroughly examined and well substantiated.

### Identified HCI Elements That Influence CDSS Functionality and Performance

#### Overview

As discussed in the previous subsections, regarding our explicit question about HCI or user perspective elements with the potential to impact CDSS functionality, we identified 12 elements after a full-text study of 43 selected papers. These elements were categorized based on their inherent nature, application context, and significance to the UX, particularly from the perspective of medical staff. As illustrated in Table 1, these elements address different HCI concerns. Although HCI elements such as “interface” inherently encompass subelements such as “visibility,” “user control,” and “data entry,” these subelements have been presented independently to highlight their unique roles and distinct contributions to CDSS functionality and usability; for example, “data entry” focuses on minimizing input errors through standardized workflows, whereas “visibility” prioritizes intuitive data presentation—distinct concerns that have been highlighted in studies on clinical efficiency and user trust [60,88].

**Table 1:** Extracted human-computer interaction (HCI) elements and insights.

HCI elements and topics and addressed concerns		References
**Alerts**		
	Usability flaws	[43,44]
	Alert fatigue	[7,45-47]
	Alert design recommendations	[48-53]
	Barriers (too many alerts)	[54]
**Ease of use**		
	Intention to use	[55]
	Understandable	[56]
	Customizability	[57]
	User-friendly features	[58]
	Jargon avoidance	[59]
	Consistency of operations	[60]
	Goal-directed design	[61]
	Facilitators (automating data fetching)	[62]
**Interface**		
	Acceptability	[63]
	Information recall issues	[64]
	Data presentation	[65]
	Visualization	[66]
	Interface features	[67]
	Physical layout	[68]
	Facilitators and barriers to presenting diagnostic information	[69]
	Heuristic semantic tags (to improve interface design)	[70]
**Visibility**		
	Error visibility	[71]
	Patient status visibility	[72]
	Importance-based highlighting	[73]
**Simplification**		
	Removing extraneous information and procedures	[74]
	Resolving the conflict between system simplicity and usefulness	[75]
**User satisfaction**		
	Users’ expectations and learnability	[76]
	Effectiveness, efficiency, and accessibility	[77]
	Barriers (mismatch between meaningful-use criteria and physicians’ expectations)	[78]
**Explainability**		
	Transparent and informative recommendations	[79]
**Flexibility**		
	Ability to change order and revise data entry	[80]
**User control**		
	Controllability for error prevention	[81]
**Data entry**		
	Accurate and structured data entry	[82]
**Individuality**		
	Individualized and user-centered approach	[83]
**Mental effort**		
	Cognitive load	[84]

This categorization of HCI elements aligns with the International Organization for Standardization (ISO) 9241-11 framework [89], which defines usability as a combination of effectiveness, efficiency, and satisfaction in achieving specific goals within a particular context. We found some studies (3/43, 7%) that directly evaluated CDSSs to assess “user satisfaction,” focusing on the outcomes of user-system interaction. These studies align with the satisfaction component of the ISO 9241-11 standard, emphasizing users’ subjective experience and acceptance of the system. In comparison, “visibility” supports the effectiveness dimension of this standard by ensuring that users remain informed about the system’s status in a timely manner, facilitating accurate decision-making. Conversely, “ease of use” corresponds directly to the efficiency dimension of ISO 9241-11 because it minimizes the cognitive and physical effort required to interact with the system, enabling users to achieve their goals with minimal waste of resources or time. Moreover, “explainability” enhances effectiveness by improving users’ understanding of system outputs, while “alerts” contribute to both effectiveness and efficiency, ensuring that critical information is promptly communicated without overwhelming users. Another HCI element, “user control,” also strengthens effectiveness by aiding error prevention and recovery, allowing users to rectify mistakes and maintain workflow reliability. As the “interface” element impacts decision accuracy and interaction speed, it is strongly related to effectiveness and efficiency. “Flexibility” and “individuality” primarily support satisfaction because they enable system adaptability to different user needs and preferences, fostering trust and engagement. “simplification” and “mental effort” impact both efficiency and effectiveness because they reduce cognitive load, eliminate unnecessary complexity, and optimize workflow processes. Another HCI element, “data entry,” contributes to accurate information for decision-making (effectiveness) and streamlines the input process to save time and reduce effort (efficiency).

The rationale for this categorization is clarified in Table 1, which outlines the main concern addressed by each study and explains how it relates to a specific HCI element. The subsections that follow provide a detailed explanation of each HCI element listed in the table.

#### Alerts

##### Overview

Alerts have been identified in this study as a major HCI element. Alerts in CDSSs are essential for enhancing patient safety and clinical efficiency. They provide immediate, actionable information to health care providers. They can warn about potential drug interactions, highlight critical patient allergies, or flag abnormal laboratory results [48]; for instance, an alert might notify a physician about a dangerously high potassium level in a patient, prompting immediate intervention. These alerts help prevent errors, ensure timely responses, and support adherence to clinical guidelines, ultimately improving patient outcomes and streamlining clinical workflows [90].

To explicitly investigate alert-related concerns within CDSSs, it is crucial to differentiate between alerts and reminders because conflating these elements can hinder effective system design. In the context of HCI within CDSSs, alerts and reminders should be discussed separately, given their distinct functions [91]. Alerts are immediate notifications requiring urgent attention, supporting real-time decision-making with minimal cognitive load. Reminders are persistent prompts designed to ensure that critical tasks are not overlooked, thereby improving adherence to clinical guidelines. They are defined as a communication or message to ensure that physicians remember critical tips [92]. Reminders can cause cognitive overload and frustration if they are too frequent or irrelevant—in such cases, they are categorized as barriers. Conversely, when reminders assist physicians, they are considered facilitators [93]. This distinction ensures a more effective optimization of HCI elements in CDSSs, balancing support and usability.

The findings from this SLR demonstrate that 31% (13/43) of the selected papers addressed alert-related topics, while Figure 3 shows that this element was mostly studied between 2011 and 2016 (11/13, 85%), although some studies (2/13, 5%) have been conducted in recent years (2020 and 2022). Research on alerts has focused on 3 main areas. Table 1 presents the studies that have addressed these categories, which are detailed in the following subsections.

##### Alert Fatigue

Alert fatigue poses a significant challenge for CDSS instruments because users may begin to neglect or override activated alerts due to their frequent occurrence [94]. In 2016, Gong and Kang [47] presented five solutions to address alert fatigue: (1) augmenting alert specificity, (2) tiering alerts based on severity, (3) applying human factors principles (such as format, content, legibility, and color), (4) customizing alerts based on patient attributes, and (5) providing tailored alerts for medical practitioners.

The timing of alerts is crucial because they should be presented at moments conducive to informed medical decision-making. It is important to strike a balance: alerts should be intrusive enough to capture attention, but they should not distract or divert users from the primary care pathway [45]. Maintaining this balance prevents alert fatigue, sometimes referred to as habitual override.

##### Usability Flaws

Usability flaws relevant to CDSSs that diminish alert performance have been identified as follows [43]:

- The distinguishing visualizations do not illustrate the variety of severity levels in the alerts.
- The information presented within alerts is dense and lacks brevity.
- A low signal-to-noise ratio, reflecting a high proportion of erroneous alerts compared to correct ones, often results from nonupdated or incorrect data.
- Alert content issues stem from missing information relevant to alert goals, data interpretation, or necessary practical recommendations.
- There is a scarcity of transparency regarding the reasons that have led to the triggering of the alert.
- The clinicians are not directed at fixing the problem identified by the alert.
- Adaptability issues point out the insufficient adjustability of the system to accommodate all types of users.
- Workload issues arise when too many tasks need to be performed to correct errors or obtain the required information.

In medical information systems, 2 main types of alert flaws are commonly observed: general and specific. General alert flaws occur when alerts lack specificity or fail to distinguish between different severity levels [44]. Specific alert flaws arise when alerts do not adequately address individual patients’ or health care providers’ unique contexts or needs, leading to ineffective or irrelevant notifications [95].

##### Alert Design Recommendations

Alert design recommendations have been addressed across the following aspects to enhance alert effectiveness (Textbox 3). Enhancements in alert functionality within CDSSs can optimize clinical workflows, ensure timely access to relevant information, mitigate alert fatigue, foster user trust, and ultimately contribute to more informed and effective clinical decision-making processes [68].

**Alert design recommendations.**

- The visual presentation of alerts can be improved by using different colors, bullet points, and clear textual guidelines [48].
- Alerts should be triggered at appropriate points within the clinical workflow and should align with real-world practices [50].
- False alarms that can occur when the alert’s logic is flawed or not based on updated information must be avoided. In some cases, rigid sensitivity calibrations can result in false alarms [49].
- The presentation of alerts should be determined based on their level of importance; for instance, higher-priority alerts may be presented in a way that interrupts the current workflow [50].
- Habituation pertains to repeated exposure to insignificant alerts. This underscores the importance of reducing the occurrence of false alarms, along with adopting an alarm philosophy aimed at minimizing alert overrides [49].
- Consistent terminology emphasizes the use of standardized and predefined words and expressions. This enhances the ability to locate specific words or data on the screen through visual screening and promotes uniformity in the generated data irrespective of geographic location [96].
- Mental models refer to an individual’s interpretation of a specific matter that influences their reaction when an alert is triggered; for instance, the color red typically evokes an immediate association with “STOP” [49].
- The content of the alert—the reason for its activation and potential medical consequences—should be kept concise [97], with additional details accessible through related data links [98].
- Font style and size can be used to convey the importance and prioritization of the alert [51,99].
- Alert visibility is a critical HCI component that should be considered in the design of alerts within the CDSS environment [52]. The alert’s dimensions as displayed on the screen (target size), luminosity, background contrast, and typography attributes should be carefully considered to quickly capture the user’s attention [100,101].
- The degree of workflow interruption must be proportional to the severity of the medical issue that triggered the alert [51]. For high-priority alerts, it may be necessary to interrupt the workflow to prompt immediate user intervention [52].
- To ensure the proximity of decision-making components, decision support tools must be incorporated within the alert; for example, the alert should include hyperlinks to medical reference websites [53].

#### Excessive and Unnecessary Alerts

Particularly in specific patient populations or under uncommon circumstances, excessive and unnecessary alerts represent a significant barrier to the effective use of alerts within CDSSs. This obstacle can be resolved by grouping similar alerts into a single notification and tailoring alert thresholds and delivery methods based on patient condition and clinical context [54].

#### Ease of Use

Ease of use was identified as a principal user concern in 19% (8/43) of the selected papers. This element is foreseeable and represents a standard expectation among users, particularly physicians. In this respect, systems should be designed so that users easily understand how to operate the system. This is referred to as perceived ease of use. A positive perceived ease of use is associated with an increased intention to use (IU) [55].

The concept of consistency will augment a system’s ease of use. It can be achieved in 2 distinct areas: jargon-free language and consistent operative patterns. Consistent terminology and operation across all system components minimize user confusion and cognitive load, enabling users to quickly and effectively achieve their goals [59].

In essence, a system that is easy to use enhances learnability because users can understand and remember operational procedures more quickly [102].

In a CDSS environment, ease of use can significantly boost system functionality by ensuring that health care professionals can quickly and without complexity access and apply clinical guidelines and decision aids [58]. In this regard, a key facilitator is automated data fetching, which streamlines the retrieval of relevant information, minimizing complexity and allowing users to focus on patient care [62].

Moreover, goal-directed design is a user-centered approach that prioritizes understanding user needs and goals throughout the design process. By engaging end users throughout the design process, goal-directed design ensures that the system effectively supports their tasks and contexts. This alignment directly contributes to ease of use by streamlining interactions and enhancing UX [61].

#### Interface

Interface, another important HCI element**,** was addressed in 19% (8/43) of the selected papers, especially between 2015 and 2021. While interface design inherently encompasses various HCI elements, the analyzed papers offer unique insights that extend beyond the scope of other discussed elements. These papers provide specific recommendations for interface improvements that can significantly enhance user interaction by optimizing system design and fostering intuitive use.

The interface of a CDSS directly influences acceptability by determining how intuitively users can access and act on clinical information. When the interface aligns with users’ workflows and preferences, it increases their willingness to adopt and use the system effectively [63]. Despite recent advances in computerized technologies, the defective design of graphical UIs in clinical settings may lead to frustration among physicians [10]. In the context of CDSSs, the UI refers to the graphical or visual representation through which users interact with the system to access, input, and interpret information [63]. An effective UI should be intuitive, user-friendly, and tailored to health care professionals’ specific needs and workflows [64].

Via integration with EHRs, well-designed interfaces within CDSSs can present a list of possible diagnoses associated with medical symptoms, the compatibility percentage of the patient’s current medical status, and the factors contributing to the diagnosed illness [65,103]. In addition, effective data visualization enables quick comprehension of complex information, promoting time efficiency by enabling health care professionals to rapidly interpret data without the need for extensive analysis [66].

A responsive and interaction-enabled UI encompasses 2 distinct features: presentation and placement. In terms of presentation, elements such as simplicity, appropriate font size, meaningful colors, acceptable contrast, and bold text enhance readability and user engagement. Regarding placement, information should occupy a prominent position, be localized for easy access, and use multiple presentation layers to facilitate quick comprehension. Together, these interface features facilitate the seamless flow of essential medical data to decision makers [67].

In the interface realm, the physical layout concept refers to the arrangement and organization of screen objects with which users interact. This layout significantly impacts user control, allowing health care professionals to directly manipulate the screen’s objects (such as buttons, sliders, and forms). When users have clear control over screen objects, they can more effectively navigate the system, customize their workflow, and respond to patient needs promptly, ultimately improving overall usability and satisfaction with the CDSS [68].

There are some facilitators and barriers relevant to the interface within CDSS environments. One of them is the content-based facilitator that enhances interface usability by presenting clear, concise, and relevant information. In this respect, tailoring content to meet the health care professionals’ specific needs and preferences and providing real-time updates on clinical guidelines will enhance the interface. Conversely, inconsistent content within the data presentation and guidelines, potentially leading to confusion and misinterpretation, has been identified as an interface barrier [69].

One reviewed paper introduced the concept of “heuristic tags” to enhance CDSS interface design [70]. The paper describes a container comprising 14 HCI elements relevant to CDSS environments, referred to as semantic tags [70]. These elements are outlined in Table 2.

**Table 2:** Semantic tags.

Tag	Description
Consistency and standards	All system providers throughout the design must adhere to a unified protocol by maintaining consistency in terminology, sequence of actions, and data localization.
Visibility of the system status	Users should be notified of ongoing processes via suitable alerts and feedback mechanisms.
Match between system and world	Users’ perceptions of the system should align with their mental models of how the system is expected to function.
Minimalist design	Any unnecessary information should be eliminated because it acts as a distraction and impedes efficiency.
Minimize memory load	Users should not be required to memorize extensive information to perform routine tasks.
Informative feedback	Users must receive informative feedback regarding their activities.
Flexibility and efficiency	The system should provide resiliency so that users can customize settings and expedite their tasks.
Good error messages	Messages should provide sufficient information for users to comprehend the nature of errors, learn from mistakes, and take corrective action.
Prevent errors	The design should deter errors by preventing incorrect actions.
Clear closure	Users must be explicitly informed when a task has been completed.
Reversible actions	Users should be allowed to recover from errors made by them.
Use user’s language	The system language must be comprehensible to the targeted users.
Users in control	Users should not feel in control of the system without encountering unforeseen circumstances.
Help and documentation	The system should offer various forms of help, such as contextual assistance, mission-focused guidance, and alphabetically organized (lexicographically arranged) help topics for easier navigation.

Therefore, interface quality will influence the precision of medical decisions by facilitating intuitive access to relevant information and enhancing user interaction.

#### Visibility

Visibility was addressed in 7% (3/43) of the selected papers, emphasizing that quick access to information requires data to be readily accessible within a short time frame and ensuring that medical data are available at expected locations. This includes the clear and immediate visibility of possible errors or system warnings, allowing users to identify and address issues quickly [71]. Within the treatment cycles pertinent to CDSSs, visibility refers to the clarity and transparency of the workflow stages and progress, meaning that health care providers can easily track and understand the status of the treatment process, including diagnostic tests, medication administration, and patient outcomes [72]. In addition, by visually emphasizing critical information such as high-risk alerts or abnormal laboratory values through color coding, bold text, or other visual cues, clinicians can quickly identify and prioritize important information [73]. As visibility criteria provide a clear and transparent view of the treatment cycle workflow, they enable users to easily monitor the progress of patient care and make informed decisions based on the current stage of treatment, leading to the best possible decision [73].

#### Simplification

Of the 43 selected papers, 2 (5%) addressed simplification elements. Simplification can be achieved by eliminating unnecessary complexities and providing an optimized workflow congruent with real-world practices [104]. Removing extraneous information and procedures is crucial for achieving this goal [74]. Pantazi et al [75] propounded a theory describing a paradox between simplification a medical system and its usefulness: systems with high usability typically can solve trivial problems, whereas solving intricate problems requires an appropriate level of complexity, which can reduce overall usability.

**Figure 4:**
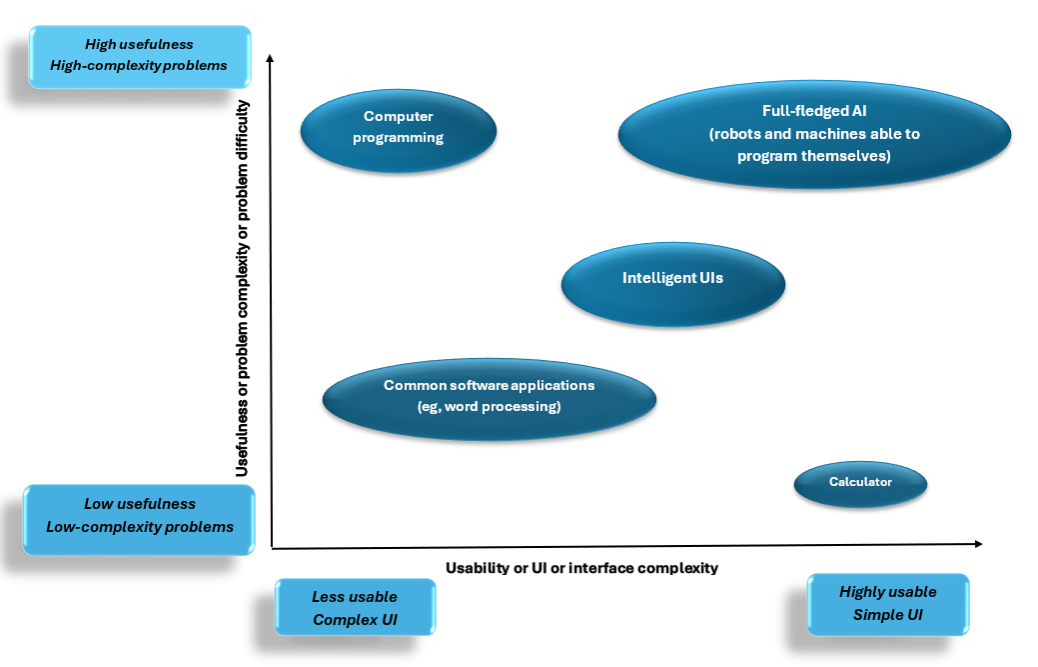
The relationship between system complexity and usability. AI: artificial intelligence; UI: user interface.

As shown in Figure 4 [75], some CDSS applications designed for intricate problems have complex UIs with lower usability levels, whereas sections with simpler structures within CDSSs, such as medical calculators, exhibit higher usability levels. Meanwhile, system designers are striving to develop medical AI systems that simultaneously enhance both usability and problem-solving capacity.

Nonetheless, while striving for complexity in medical procedures can be inherently beneficial, it is crucial to maintain a balance between usability and necessary intricacy [105]. Striking this balance ensures that procedures are not unnecessarily complex, saves time, and ultimately enables rapid emergency medical decision-making [106,107].

#### User Satisfaction

User satisfaction was discussed in 7% (3/43) of the selected papers. Recognized as a central focus in HCI research, user satisfaction is paramount to the effectiveness of CDSSs. This concept encompasses aspects such as system acceptability, learnability, memorability, and accessibility [76].

UCD methods are essential for achieving high user satisfaction [108]. By prioritizing the user’s needs and perspectives throughout the development process, CDSSs can be tailored to meet their specific requirements [109]. Learnability facilitates users in quickly understanding how to operate the system, which enhances initial engagement [110]. Memorability ensures that the system’s functions are easily recalled once learned, reducing the need for repeated training [111]. Accessibility ensures that the required medical information and history are promptly accessible in the system [77]. Incorporating these elements boosts overall user satisfaction, improves efficiency and adoption, and ultimately leads to better patient outcomes in clinical environments.

Conversely, the mismatch between meaningful-use criteria and physicians’ expectations represents a significant barrier to user satisfaction within CDSSs. In these cases, the requirements set by system designers do not align with physicians’ practical needs and processes, leading to frustration and decreased usability. This misalignment can hinder the effective adoption of CDSSs because physicians may find the systems cumbersome or irrelevant to their clinical workflow. Addressing this gap is essential for improving user satisfaction and ensuring that CDSSs effectively support health care professionals in delivering high-quality patient care [78].

#### Explainability

Explainability, as identified in 2% (1/43) of the selected papers, is a critical focus in the ongoing discourse surrounding CDSSs and medical AI [112]. Transparency, a cornerstone of explainability, fosters trust between the user and the system [113]. Once the CDSS provides recommendations and solutions, physicians can understand why and how the advice was generated. This understanding augments their clinical expertise and contributes to more informed decision-making over time [79].

#### Flexibility

Flexibility was articulated in 2% (1/43) of the selected papers. This element requires significantly more attention and could be leveraged to enhance CDSS functionality. Adjustable medical systems typically achieve higher adoption rates, particularly when they meet physicians’ need for flexibility [114]. In this context, 3 diverse dimensions related to flexibility concerns have been identified [80]: (1) user tendency to return to previous stages to correct or modify information; (2) data entry in multiple different sequences; and (3) customizable data presentation.

Flexibility is a crucial attribute when designing digital pathways. By aligning flexibility with actual clinical workloads**,** treatment routes can be tailored to meet physicians’ and patients’ specific needs and demands [115]. This alignment enhances medical decision accuracy, enhances user satisfaction, and ultimately improves patient outcomes [116].

#### User Control

User control was addressed in 2% (1/43) of the selected papers, emphasizing the importance of instructions that align with real-world workflows, aiding users in recovering from and preventing errors. Interfaces that are compatible with established practices help users rectify mistakes and reduce the recurrence of common errors, enhancing overall usability and efficiency [81]. In this context, an important framework is the unified theory of acceptance and use of technology (UTAUT). UTAUT is used to predict and understand individuals’ acceptance and adoption of new technologies, integrating various factors such as performance expectancy, effort expectancy, social influence, and facilitating conditions. By enabling users to recover from mistakes and easily control their interactions, systems can enhance user satisfaction and increase the likelihood of technology adoption, as predicted by the UTAUT model [117].

#### Data Entry

Data entry was investigated in 2% (1/43) of the selected papers, with a focus on designing systems that minimize potential user errors [82]. Structured data entry management is fundamental in CDSSs, ensuring organized, standardized, and systematic data input. Emerging technologies such as voice recognition technology and natural language processing have facilitated and improved the data entry aspect in eHealth systems. These advancements minimize errors and inconsistencies, ultimately enhancing the reliability and effectiveness of CDSSs in supporting clinical decision-making [118].

#### Individuality

Individuality was presented in 2% (1/43) of the selected papers. In this area, Klumpp et al [83] discuss the HCI concept as a theory encompassing 4 key issues that shape individuals’ interactions with computerized systems. The first factor is the identification of technology, including all technological features applied to the system [119]. The second factor is the assignment, referring to a defined task where the technology used varies depending on the satisfaction and aims of the task [120]. The third factor is context, which can vary depending on the geographic, corporate, or community conditions, meaning that the recommendations should be customized according to the geographic positions and other local conditions [121]. The fourth factor involves human dimensions, such as population characteristics, cognitive abilities, and the attitudes of individuals [122]. In other words, customizability should be based on the intended populations’ approaches, attributes, and desires. Concentrating on system individualization and customization can enhance the accuracy of decision-making in CDSSs [83].

#### Mental Effort

Mental effort was identified as an HCI element in 2% (1/43) of the selected papers. The Rating Scale Mental Effort (RSME), first proposed by Militello et al [84], is designed to accurately measure perceived mental effort during task completion. In other words, it measures the extent to which individuals feel they have exerted mental effort to complete a task. The RSME can be used to gauge how effectively CDSSs aid medical practitioners without overly complicating their decision-making processes. A low RSME score indicates that the CDSS is well integrated into clinical workflows, and CDSS functionality is enhanced without imposing significant additional cognitive complexity on the user [123].

## Discussion

### Principal Findings

While several studies have explored HCI elements in CDSS environments, each has focused on specific aspects independently. This has left a research gap regarding a comprehensive analysis and categorization of these effective elements. This study attempts to present a scientific framework for CDSS design by identifying HCI elements that influence CDSS functionality across different dimensions. In this respect, after reviewing 43 selected papers in this SLR, we identified and stratified 12 key HCI elements, thereby distinguishing our investigation from the selected studies, which, although they explored the identification and classification of HCI elements, were experimental in nature, with a limited focus on specific, monitored elements.

A common thread among the selected studies is their focus on HCI elements relevant to CDSS applications, which can substantially influence CDSS functionality and performance. Throughout this SLR, the categorization of studies under specific HCI elements depended on the primary focus of each study. While some studies explicitly investigated a single HCI element, others addressed multiple aspects (such as heuristic semantic tags [70]) or offered broad recommendations. In such cases, the main HCI concern emphasized in the study was the selection criterion. This approach ensured that each study was categorized based on its core usability focus, maintaining consistency and relevance in the analysis.

Throughout this SLR, the categorization of studies under specific HCI elements depended on the primary focus of each study. While some studies explicitly investigated a single HCI element, others addressed multiple aspects (such as heuristic semantic tags [70]) or offered broad recommendations. In such cases, the main HCI concern emphasized in the study was the selection criterion. This approach ensured that each study was categorized based on its core usability focus, maintaining consistency and relevance in the analysis.

The interface is considered a distinct HCI element because it serves as the primary medium for user interaction [124]. Although it encompasses visibility, data presentation, and ease of use, its role transcends these components [125,126]. Indeed, the interface functions as the platform that enables the delivery and experience of these elements, making it a distinct and critical component of the HCI context [127]. This distinction is crucial for CDSS environments, where the interface must accommodate complex clinical workflows and user needs to ensure usability and patient safety. Hence, despite its broad scope, the interface has been examined as an independent HCI element in some studies, allowing for a more focused and in-depth evaluation of its inherent contributions to UX and decision-making in CDSS environments [128]. Thus, we considered it a separate HCI element in this study.

The relationship between HCI and UI and UX design further underscores the importance of the interface as an independent element. While UI focuses on the visual and interactive components of a system (eg, buttons, menus, and layouts) and UX encompasses the user-centered experience (including usability, accessibility, and emotional satisfaction), the interface concept (within HCI) is the physical manifestation of these principles [129,130]. As Paneru et al [131] explain, the interface is a visual and interactive layer that enables users to interact with digital products. It is the medium through which all other HCI elements are experienced, making it a foundational component of HCI. However, the interface also extends beyond traditional UI and UX design by incorporating considerations such as workflow alignment, cognitive load reduction, and task efficiency, which are central to HCI. By contrast, Motlagh and Safaei [132] emphasized that, rather than relying solely on UI and UX considerations, HCI evaluation in health care systems should prioritize error prevention, cognitive effort, and information recall. This means that HCI elements promote interfaces that support accurate decision-making and clinical workflow integration.

According to ISO 9241-11, usability measures how effectively, efficiently, and satisfactorily users can achieve their goals with a system in a particular context. This highlights the importance of context and task dependency in evaluating usability because the relevance and effectiveness of HCI elements vary across different environments and user needs [133]. The HCI elements identified in this study align with these usability dimensions but manifest differently depending on task requirements and system context. In frequently used systems such as EHRs in busy hospitals, ease of use, simplification, and task efficiency are paramount HCI elements. Clinicians using these systems need interfaces that minimize cognitive load and streamline workflows because even small inefficiencies can accumulate, leading to frustration and errors. In this context, alerts must be designed to be highly visible but nonintrusive so that they support rather than disrupt workflow [48]. In these circumstances, data entry mechanisms should be optimized for speed and accuracy because frequent use requires efficiency. Conversely, in an infrequently used system (such as a specialized diagnostic tool used only occasionally), HCI elements such as explainability become more critical. Users of such systems will need more guidance and contextual support to navigate the interface effectively. Regarding user control and flexibility, although these elements align with the satisfaction component of the ISO 9241-11 standard, their impact is also context dependent. Experienced physicians may prefer more flexible customization to streamline interactions, while novice users benefit from structured interfaces with clear navigation paths and constraints to prevent errors [80,81]. As shown in Table 1, the identified HCI elements were categorized based on their primary usability contributions; nevertheless, their actual significance in improving CDSS usability depends on the task, frequency of use, and clinical environment.

Two closely related but distinct HCI elements—ease of use and simplification (simplification)—play unique roles in enhancing CDSS usability. Ease of use refers to the system’s ability to provide an intuitive and seamless interaction experience, minimizing cognitive and physical effort for users. It encompasses aspects such as jargon avoidance, consistency of operations, and efficient task execution, ensuring that clinicians can navigate the system with minimal frustration [56,58-60]. By contrast, simplification focuses on reducing unnecessary complexity within the system, such as streamlining workflows**,** eliminating redundant steps, and minimizing information overload [74,75]. While ease of use improves UX, simplification optimizes system design by removing obstacles and extraneous procedures that could hinder task completion. Moreover, ease of use is closely tied to user satisfaction and IU. An easy-to-use system facilitates navigation, reduces cognitive efforts, and leads to a more positive UX, ultimately increasing IU and user satisfaction.

This study recognized another prevalent and critical challenge faced by CDSS designers: balancing the paradox between system simplification and maintaining its usefulness in solving complex problems, even if doing so leads to increased complexity. This balance is essential for developing systems with high usability and appeal, ensuring that users can effectively interact with the system without being overwhelmed by its complexity [75]. To reach this equilibrium, designers must adopt a user-centered approach that prioritizes the needs and capabilities of health care professionals. This involves continuous user testing and feedback loops to refine the interface and functionality [59]. In addition, incorporating adaptive interfaces can reduce complexity based on the user’s expertise and the specific task to help manage this paradox, resulting in augmented overall satisfaction and higher adoption rates [65].

This study attempted to highlight the HCI elements dedicated to CDSSs, specifically those that address user requirements across various dimensions such as informative notifications [52], aesthetics [61,71], contextual facets [79], unification [70], and psychological aspects [84]. To illuminate all HCI aspects within CDSSs, this study also identified elements that represent challenges in this domain requiring mitigation, such as existing HCI barriers [78], usability flaws [43], user fatigue [7], and the phenomenon of alert override [49]. Although considering the identified HCI aspects is crucial, the final goal is to improve user interaction with the system, enhance ease of use, and anticipate and fulfill user expectations. This approach fosters user satisfaction and promotes a positive UX [74]. This focus on users as major “players” in the design process is now an established principle. Hence, UCD methods are fundamental to the development of CDSSs with high levels of user interaction [134]. Depending on the system’s complexity and ease of use, both user interaction with the system and data accuracy can vary, consequently affecting the accuracy of medical decision-making [58].

In summary, this study provides comprehensive insights into the essential HCI elements applicable in the CDSS environment, answering the RQs. It contributes to the field by systematically categorizing these elements and developing a structured framework aimed at improving usability and medical decision-making outcomes.

### Conclusions

Of the 923 papers found in 3 databases (PubMed, Scopus, and Web of Science), 43 (4.7%) met the predefined criteria with regard to addressing HCI elements applicable in CDSS environments. This SLR indicates that attention to HCI elements tailored to CDSSs has increased since 2015. In this study, we identified 12 HCI elements. While elements such as “ease of use,” “interface,” and “alerts” have consistently garnered attention from experts, others such as “user satisfaction,” “visibility,” and “explainability” have recently received increased focus. This SLR has answered the RQs regarding the HCI elements that can affect CDSS functionality and described how these elements impact CDSS performance. Moreover, the study categorized the identified aspects according to their context and scientific perspectives.

This study identified a gap in current research: the lack of a comprehensive consideration of HCI elements within CDSS design. By leveraging the findings from this investigation, a set of applicable HCI elements for CDSS environments can be established. Consequently, these findings have the potential to enhance the synergistic effect between medical information systems and CDSSs, ultimately leading to improved overall performance.

This research provides the groundwork for generating more structured and reliable datasets to support medical decision-making. Systems designed with these HCI elements are expected to reduce missing data, minimize data redundancies, and improve data clarity. Accordingly, the accuracy and reliability of medical decisions derived from these systems will be noticeably enhanced. Future studies can leverage this framework to develop and examine CDSS systems that improve user interaction and ensure higher data integrity and precision in medical decision-making. This structured approach will advance medical informatics, leading to more efficient, accurate, and user-friendly CDSS solutions.

## Appendix

Multimedia Appendix 1 PRISMA (Preferred Reporting Items for Systematic Reviews and Meta-Analyses) checklist.

Multimedia Appendix 2 The raw data from this study, including extracted papers from Scopus (506/923, 54.8%), Web of Science (188/923, 20.4%), and PubMed (229/923, 24.8%).

Multimedia Appendix 3 The final set of 43 papers selected for analysis.

## Abbreviations

AI: artificial intelligence
CDSS: clinical decision support system
DSS: decision support system
EHR: electronic health record
EMR: electronic medical record
HCI: human-computer interaction
ISO: International Organization for Standardization
IU: intention to use
MQ: mapping question
PRISMA: Preferred Reporting Items for Systematic Reviews and Meta-Analyses
RQ: research question
RSME: Rating Scale Mental Effort
SLR: systematic literature review
UCD: user-centered design
UI: user interface
UTAUT: unified theory of acceptance and use of technology
UX: user experience
